# Diabetes self-care practice and associated factors among type 2 diabetic patients in public hospitals of Tigray regional state, Ethiopia: A multicenter study

**DOI:** 10.1371/journal.pone.0250462

**Published:** 2021-04-21

**Authors:** Goitom Molalign Takele, Medina Abdulkadir Weharei, Hiyab T/Michael Kidanu, Kahsu Gebrekirstos Gebrekidan, Birhan Gebresillassie Gebregiorgis

**Affiliations:** 1 Department of Emergency and Critical Care Nursing, School of Nursing, College of Health Sciences, Mekelle University, Mekelle, Ethiopia; 2 Department of Pediatrics and Child Health Nursing, School of Nursing, College of Health Sciences, Mekelle University, Mekelle, Ethiopia; 3 Department of Nursing, College of Health Sciences, Debre Berhan University, Debre Berhan, Ethiopia; University of Illinois at Urbana-Champaign, UNITED STATES

## Abstract

**Background:**

The prevalence of type 2 diabetes is increasing steadily at an alarming rate. Ethiopia is placed fourth among the top five countries of the African region members of the international diabetes federation. This study aimed to determine the level of diabetes self-care practice and associated factors among patients with type 2 diabetes mellitus attending public hospitals of the Tigray region.

**Methods:**

An institution-based, cross-sectional study was conducted in six selected hospitals of Tigray region from January to February 2020. Study participants were recruited using a systematic random sampling method. Diabetes self-care practice was assessed using Summary Diabetes Self-Care Activities (SDSCA) assessment tool. The data were collected by trained nurses via face-to-face interview. Binary and multivariable logistic regression analyses were used to identify factors associated with self-care practices. Statistical significance was declared at p-value < 0.05.

**Results:**

A total of 570 patients with type 2 diabetes were included in this study. The mean (SD) age of the participant was 46 (±14.6) years. Less than half (46.7%) of the participants had good diabetes self-care practices. Surprisingly, only 68 (11.9%) of the participants had access to a personal glucometer. Urban residency (AOR = 1.9, 95% CI = 1.20–2.94), age group 48–63 years (AOR = 2.1, 95% CI = 1.19–3.98), not having a formal education (AOR = 2.6, 95% CI = 1.32–5.25), having family support (AOR = 1.9, 95% CI = 1.24–2.85), and having a personal glucometer at home (AOR = 6.1, 95% CI = 2.83–13.0) were the factors associated with good diabetes self-care practices.

**Conclusion:**

The diabetes self-care practice in the region was found to be poor. Where factors like, being an urban resident, age group between 49–63 years, not having a formal education, and having a personal glucometer at home were associated with good self-care practices. Health care providers might have to consider actions to act on the identified factors and improve the level of self-care practices of the patients.

## Introduction

Diabetes Mellitus (DM) is one of the fastest-growing global health emergencies of the 21^st^ century [1, 2]. According to the International Diabetes Federation (IDF) report in 2019, an estimated 463 million adults aged 20–79 years worldwide have diabetes and this figure is projected to be 578.4 million, and 700.2 million by 2030 and 2045 respectively [2]. Ethiopia is placed fourth among the top five African member countries of IDF (32 countries), having 1.7 million people with diabetes (age 18–99) [2].

Diabetes self-care practice includes physical activity, self-monitoring of blood glucose, adequate nutrition, foot care, and adherence to medications [3]. Self-care practices remains the mainstay management of diabetes, as the majority of the disease management is carried out by patients themselves or their families [4]. Diabetes care is complex and more demanding, which needs a better understanding of the disease beyond monitoring blood glucose levels [5]. Therefore, the cornerstone of managing type 2 diabetes mellitus (T2DM) is a healthy lifestyle, including a healthy diet, regular physical activity, not smoking, and maintaining a healthy body weight [6, 7].

Even though, adherence to diabetes self-care practice has shown a remarkable reduction in the incidence and progression of DM complications [8], different studies in Ethiopia showed that it is poorly practiced [9, 10]. Previous studies showed that, factors like duration of diabetes (early years), younger ages, educational status, attending diabetes education, living in a rural area, male gender, lack of family support, having comorbidities, poor knowledge about diabetes, and lack of self-monitoring glucometer, were found to affect diabetes self-care practices [9–12].

Although it is known that diabetes self-care practice is vital in the management of the disease, the magnitude and factors associated with self-care practices are not well studied at the regional level. Therefore, this study aimed to assess the level of self-care practices and associated factors among T2DM patients attending government hospitals in the Tigray region.

## Method

### Study setting and period

An institution-based cross-sectional study was conducted from January to February 2020, in six public hospitals of Tigray regional state namely: “Wukro general hospital”, “St. Marry general hospital”, “Sihul general hospital”, “Lemlem Karl general hospital”, “Ayder comprehensive specialized hospital”, and “Axum university referral hospital”. Administratively, the region is divided into 7 Zones, and those selected hospitals found in six of these seven zones. There are two specialized hospitals and 15 general hospitals in the region. According to the available most recent population census of 2007 the region has an estimated population of more than 5 million. Majority (80.5%) of the population live in rural areas and the majority of them were Orthodox Christian by religion.

### Sample size calculation and sampling method

The sample size was calculated using single population proportion formula assuming a 95% confidence interval, 5% margin of error (d), and the 50.2% proportion of T2DM patients with a good level of diabetes self-care activities, from a study conducted in the southwest of Ethiopia [13]. By considering a design effect of 1.5, the total sample was 576. The inclusion criteria’s were age ≥18 years, diagnosed with T2DM, able to understand the local language “Tigrigna”, and had follow-up for at least six months. Patients diagnosed with gestational diabetes or mental disorders were excluded. A multistage sampling method was used to reach the study hospitals. First, five zones out of the seven in the region were selected. Followed by selecting six (2 specialized and 4 general hospitals) out of the 2 specialized and 12 general hospitals within these zones. Finally, the study samples were recruited using the systematic random sampling method from the selected hospitals. The sample was allocated proportionally to each hospital i.e. 155 to ACSH and Axum referral hospitals each, and 69 each to the remaining hospitals based on the number of patients on follow-up visits during the data collection period. Study participants were selected using a systematic random sampling method of every 2–4 patients in all hospitals during their visit to the DM clinic.

### Data collection procedure and tool

The English version standardized instruments were translated into the local language “Tigrigna” and then translated back to English. The tool contains information on socio-demographic, clinical characteristics, Summary of Diabetes Self-Care Activities (SDSCA) instrument [3], and the Diabetes Knowledge Test (DKT) [14, 15]. SDSCA is a self-report measure with four components of diabetes self-management (diet, exercise, blood sugar testing, and foot care). The respondents were asked to rate how many days during the past 7 days did they performed a specific self-care behavior. The scale ranges from 0 to 7, whereby higher scores correspond to higher diabetes management activities. A mean score was calculated for each domain (diet, exercise, blood glucose testing, foot care, and smoking), whereby the scores were categorized as “good” for scores above mean value and “poor” for scores less than the mean value. The overall mean score was calculated by summation of the mean score each for diet, exercise, foot care, and blood glucose testing divided by four [9]. After calculating the overall mean score, it was classified as having “good self-care practice” if the patient scored ≥ 3 or “poor self-care practice” if the patient scored < 3.

For the knowledge test, the University of Michigan Diabetes Research and Training Center, diabetes knowledge test (DKT) was used [14, 15]. The DKT is a 23-item multiple-choice test designed to assess knowledge about diet, exercise, blood glucose levels, and testing, and self-care activities have been adopted and tested [13]. Each item has three or four multiple choices with only one correct answer. The first 14 items are designed for all adults with type 2 diabetes, while items 15–23 apply only to those taking insulin [15]. Scores on the DKT were computed for each participant. The score was determined by dividing the number of correct answers by the total number of questions (23 questions for patients taking insulin and 14 for those receiving oral hypoglycemic agents). Scores ≥75%, 74–60%, and ≤59%, respectively, were labeled as high (good), acceptable (medium), and poor knowledge on diabetes [14]. The internal consistency reliability estimated with Cronbach’s α was 0.84 and 0.93 for DKT and SDSCA tools respectively.

The data were collected by trained nurses using an interviewer-administered method. The tool takes 20–30 minutes to complete.

Body mass index (BMI) Body mass index (BMI) was categorized as normal weight if BMI was 18.5–24.9, underweight if BMI was < 18.5, overweight if BMI was 25–29.9 kg/m2, and obese if BMI was ≥ 30 [16].

### Ethical considerations

Ethical approval was obtained from the Mekelle University, College of Health Sciences, Ethical review board (ERC 13179/2020). Written signed consent for participation was obtained before data collection. Participant autonomy was maintained by telling them that they can refuse or stop their participation at any time. To maintain the confidentiality of the participants no personal identifiers were used.

### Data analysis

The data were cleaned, coded, entered into Epidata.3.1, and then exported into SPSS version 25 for analysis. Descriptive statistics including mean, median, standard deviations, and range values for continuous data, as well as percentage and frequency tables for categorical data were computed. Binary logistic regression analysis was used to identify any association between the dependent and independent variables. The Crude Odds Ratios (COR) with a 95% confidence interval was estimated in the bivariate analysis to assess the association between each independent variables and the outcome variable. Variables with a p-value less than 0.25 on binary logistic regression analysis were subjected to multivariable logistic regression analysis.

In multivariable logistic regression model fitness was tested using Hosmer-Lemeshow goodness-of-fit. We checked multi-collinearity among selected independent variables via variance inflation factor (VIF) and none was found. Adjusted Odds Ratio (AOR) with a 95% confidence interval was estimated to assess the strength of the association with diabetes self-care practices. Spearman’s correlation coefficient was used to assess the association between diabetes-related knowledge and self-care practices. A p-value less than 0.05 was considered statistically significant.

## Results

### Socio-demographic characteristics of the participants

Out of the 576 participants570 of them have responded making a response rate of 99%. The mean (± SD) age of the respondents was 46 (±14.6) years, with 183 (32.1%) of them were within the age range of 34–48 years and more than half (55.8%) of them were males. Three hundred and fifty one (61.6%) of the participants were married. Regarding their educational status, 165 (29%) did not have formal education, and 104 (18.2%) of the participants had degrees and above. Five hundred and thirty seven (94.2%) of them were Tigrians and nearly two-thirds (64.7%) of them were urban residents. Three hundred fifty-one (61.6%) of the respondents had poor diabetes-related knowledge [Table 1].

**Table 1:** Socio-demographic characteristics of participants (n = 570).

Variables	Category	N (%)
Gender	Male	318(55.8)
	Female	252(44.2)
Age (in year)	18–33	126(22.1)
	34–48	183(32.1)
	49–63	185(32.5)
	>63	76(13.3)
Marital status	Single	80(14)
	Married	351(61.6)
	Divorced	98(17.2)
	Widowed	41(7.2)
Educational status	No formal education	165(29)
	Primary school	190(33.3)
	Secondary school	111(19.5)
	College and above	104(18.2)
Employment status	Employed	256(44.9)
	Unemployed	147(25.8)
	Farmer	92(16.1)
	Student	32(5.6)
	Others*	43(7.5)
Religion	Orthodox	416(73)
	Muslim	128(22.5)
	Others**	26(4.5)
Family support	Yes	323(56.7)
	No	247(43.3)
Ethnicity	Tigray	537(94.2)
	Amhara	27(4.7)
	Others	6(1.1)
Place of Residence	Rural	201(35.3)
	Urban	369(64.7)
Body mass index	<18.5 (underweight)	54(9.5)
	18.5–24.9 (normal weight)	429(73.2)
	25–29.9 (overweight)	75(13.5)
	≥30 (obese)	12(1.8)
Knowledge of diabetes	Good	65 (11.4)
	Acceptable	154 (27)
	Poor	351 (61.6)

*: retired, housewife

**: Catholic, protestant, 7^th^ day Adventist.

### Clinical characteristics of the respondents

The mean (SD) duration of DM was 6 (± 4.36) years and 316 (55.4%) of the respondents had a family history of DM. A diabetes-related complication was seen in more than two-third of participants. Of the 129 (22.6%) who had diabetes-related history of hospitalization53 (41.1%) were hospitalized twice. Surprisingly, only 68 (11.9%) of the participants have access to a personal glucometer [Table 2]. Of these who had personal glucometer majority (91.2%) of them had at least completed primary education, and aged between 34 and 48 years (37%) and 49–63 years (28%), and almost all (95.6%) of them were urban residents.

**Table 2:** Clinical and medication characteristics of the participants (n = 570).

Variables	Category	N (%)
Duration of living with DM	< 5 years	303 (53.2)
	5–10 years	193 (33.8)
	>10 years	74 (13)
Family history of DM	Yes	254 (44.6)
	No	316 (55.4)
DM related complication	Yes	402 (70.35)
	No	168 (29.5)
Having personal glucometer	Yes	68 (11.9)
	No	502 (88.1)
Smoking	Yes	36 (6.3)
	No	534 (93.7)
Alcohol drinking	Yes	63 (11.1)
	No	507 (88.9)
DM related hospitalization	Yes	129 (22.6)
	No	441 (77.4)
Number of hospitalizations	Once	72 (55.8)
	Twice	53 (41.1)
	Three times and above	4 (3.1)
Cause of hospitalization	Hyperglycemia	41 (31.8)
	Infection	42 (32.6)
	Hypoglycemia	25 (19.4)
	Others	21 (16.3)
Type of anti-diabetic drugs	Oral hypoglycemic agents	301 (52.8)
	Insulin	224 (39.3)
	Both	45 (7.9)

Regarding the diabetes self-care practice domains, more than half of the patients had poor diabetes self-care practice across all domains except for foot care (43%) [Table 3].

**Table 3:** Distribution of diabetes self-care practice domains (n = 570).

Self-care practice domains	Good % (95% CI)	Poor % (95% CI)
Diet	49.8 (45.6, 54.0)	50.2 (46.0, 54.4)
Exercise	49.8 (45.6, 54.0)	50.2 (46.0, 54.4)
Blood glucose testing	38.2 (34.2, 42.4)	61.8 (57.6, 65.8)
Foot care	56.3 (52.1, 60.4)	43.7 (39.6, 47.9)
**Over all self-care practices**	**46.7 (42.2, 50.6)**	**53.3 (49.4, 57.8)**

**Good:** mean score of ≥3 on SDSCA, **Poor:** mean score of <3 on SDSCA.

### Factors associated with diabetes self-care practice

On binary logistic regression analysis variables like age, educational status, family support, place of residency, BMI, duration of diabetes, and having personal glucometer at home were statistically associated with diabetes self-care practices. The multivariable logistic regression analysis result showed that urban resident participants were 1.9 times more likely to have good self-care practice (AOR = 1.9, 95% CI = 1.20–2.94, p = 0.006) compared to rural resident participant. The patients’ age group was also significantly associated with self-care practice: where patients with age groups between 49–63 were 2.1 times more likely to have good self-care practice than those aged 18–32 years (AOR = 2.1, 95% CI = 1.19–3.98, p = 0.011). Participants who do not have any formal education were 2.6 more likely to have good self-care practice than those who had degree and above (AOR = 2.616, 95% CI = 1.32–5.25, p = 0.006). In addition, patients with family or social support were also 1.9 times more likely to have good self-care practices than their counterparts (AOR = 1.9, 95% CI = 1.24–2.85, p = 0.003). Having a personal glucometer at home was also another factor associated with good self-care practices. Patients with a personal glucometer at home were 6.1 times more likely to have good self-care practices than patients who did not have personal glucometer (AOR = 6.1, 95% CI = 2.83–13.0, p<0.0001) [Table 4].

**Table 4:** Factors associated with self-care practices among T2DM patients (n = 570).

Variables	Category	Self-care practice		COR(95%,CI)	AOR(95%,CI)
		Good	Poor		
Age	18–33	41	84	1	1
	34–48	77	106	1.4(0.92–2.39)	1.2(0.54–2.65)
	49–63	112	72	3.1(1.97–5.13)*	2.1(1.19–3.98) *
	>63	33	42	1.6(0.89–2.90)	0.8(0.37–1.80)
Educational status	No formal education	116	49	4.8(2.86–8.26)**	2.6(1.32–5.25)*
	Primary school	82	108	1.51(0.91–2.48)	0.8(0.46–1.49)
	Secondary school	30	81	0.73(0.40–1.30)	0.6(0.31–1.15)
	College and above	35	69	1	1
Family support	Yes	196	125	2.00(1.43–2.80)**	1.9(1.24–2.85)*
	No	109	140	1	1
Residential area	Rural	73	121	3.00(2.01–4.31)**	1.9(1.20–2.94)*
	Urban	232	131	1	1
Body mass index	<18.5	11	43	0.21(0.05–0.83)*	0.2(0.05–1.00)
	18.5–24.9	198	229	0.70(0.21–2.39)	0.7(0.20–2.78)
	25–30	49	28	1.43(0.41–5.31)	2.0(0.47–8.68)
	>30	6	6	1	1
Duration	< 5 years	125	178	1	1
	5–10 years	110	83	1.12(0.67–1.91)	1.1(0.75–1.85)
	>10 years	28	46	2.13(1.24–3.74)*	0.5(0.28–1.10)
Having personal glucometer	Yes	58	10	6.00(2.98–11.93)**	6.1(2.8–13.00)**
	No	246	256	1	1

*p-value <0.05

**p-value<0.0001

AOD: Adjusted Odd Ratio, COR: Crude Odd Ratio CI: Confidence Interval. Hosmer-Lemeshow goodness-of-fit = 0.457.

Spearman’s rank-order correlation was run to determine the relationship between diabetes-related knowledge and self-care practices. There was a very week, positive correlation, which was not statistically significant (r = 0.1, p = 0.556). Similarly, even though we hypothesized there could be an association between diabetes knowledge and self-care practice, the binary logistic regression analysis result also showed no association i.e., acceptable level of knowledge (COR = 1.02, 95% CI = 0.59–1.73, p<0.941), a good level of knowledge (COR = 1.24, 95% CI = 0.69–2.22, p<0.46) compared to those having a poor level of knowledge.

## Discussion

Self-management strategies such as self-monitoring of blood glucose, dietary restrictions, regular foot care, and ophthalmic examinations have been shown to markedly reduce the incidence and progression of diabetes-related complications [8, 5]. Self-care practices and lifestyle modification remains the mainstay treatment of T2DM [5]. This study was aimed to assess the level of self-care practices and associated factors among T2DM patients in public hospitals of the Tigray region, Ethiopia.

This study showed that less than half (46.7%) of the participants had good diabetic self-care practice. This was consistent with studies conducted in the Adama, Oromia region of Ethiopia, (49.1%) [17], and southwest of Ethiopia (49.2%) [13]. However, it was higher compared to other studies conducted in Bahir Dar, northwest of Ethiopia, which was only 28.4% [10] and Harar 39% [18]. But, the finding of this study was lower than studies from India (57%) [19] and Nekemte, western Ethiopia 60.7% [9]. This difference could be due to variation in cultural, and socio-economic aspects of the society as Ethiopia is a diverse country [20]. Additionally, around one-third of participants in our study were from a rural area and they might be faced with difficulties in getting access to health care services, and opportunities for education on the management of their disease.

In the present study, self-care practice was significantly associated with the age of the participants. Where better self-care practice was seen among the age group between 49–63 years, compared to their counterparts’ age group between 18–33 years. Similarly, a study from India [19] and Egypt [20] also showed that an increase in age was associated with good self-care practices. This might suggest that the longer they stay with the disease, the more they become conscious and aware of their health and adjust their lifestyle.

This study found that those with no formal education were more likely to adhere to diabetes self-care practice. On the contrary, other studies found that the higher the educational status it is likely to have good self-care practice [10, 13, 18]. This could be because regardless of their educational status patient might adhere to self-care practices based on the information they receive from health care providers or the media. This might be also due to the social desirability bias as the questionnaire has been filled with an interviewer-administered method.

As the management of diabetes is more complex and multifaceted family and social support play an important role in patient’s treatment adherence [5]. In line with this, in this study patients with a family or social support were more likely to have good diabetes self-care practice. This was supported by other studies conducted in Gondar, Ethiopia [21], and Malaysia [22]. A study conducted in Thailand on family-oriented self-management programs also showed that engaging family support for individuals with T2DM has the potential to reduce the demands on diabetes educators and health services by providing additional support and potentially reducing complications [23] which could be due to good a self-care practice.

The availability of a personal glucometer at home was very low (11.9%). Similarly, a systematic review of sub-Saharan countries revealed on average only 15% of all patients were able to test their blood glucose level at home [11]. In the present study, the odd of good self-care practices were high among patients who have personal glucometers at home. This finding is also supported by other studies conducted in, the central zone of Tigray, Ethiopia, [12] and western Ethiopia [9].

Additionally, this study also showed that patients from urban areas were more likely to have good diabetes self-care practices compared to rural residents. This was in line with studies conducted in different parts of Ethiopia, such as Mekelle [24], Harar and Dire Dawa [18], Gondar [21], and India [25]. Previous studies have also found that, there is a misconception and poor knowledge both on the treatment and self-care practices of diabetes among patients from rural areas [26, 27]. This variation could be explained due to easy access to information through media, the internet, and the health care facilities of urban residents.

Different studies have shown that having a good or acceptable level of diabetes-related knowledge has been associated with good self-care practice [13, 22, 24]. On the contrary, our finding showed that there was no significant association between them.

### Strength and limitations

This multicenter study was conducted in six hospitals of the region and would have a better representation of the study participants and generalizability of the result. The use of multivariable logistic regression analysis would have benefit in controlling the confounding effect of variables. However, this study also has some limitations, since this was a cross-sectional study, the causal effect relationship between variables could not be established. Secondly, as the study asks the self-care activities of patients for the past seven days and there might be a recall bias among respondents. Another limitation of this study is that as the data were collected using an interviewer-administered method the responses are prone to social desirability biases.

## Conclusion

In conclusion, the level of diabetes self-care practice among T2DM patients in the Tigray region was found to be poor. Urban residency, age group between 49–63 years, having family or social support, not having a formal education, and having personal glucometer at home were predictors of good self-care practices. This suggested that clinicians and nurses might have to consider giving emphasis on caring and giving follow-up services to DM patients coming from rural areas. All stakeholders dealing with this issue should work together to close these gaps.

## Abbreviation

DM: Diabetes Mellitus
T2DM: Type 2 Diabetes Mellitus
AOR: Adjusted Odd Ratio
COR: Crude Odd Ratio
IDF: International Diabetes Federation
CI: Confidence Interval
SPSS: Statistical Package for Social Sciences
BMI: Body Mass Index
FBG: Fasting Blood Glucose
DKT: Diabetes Knowledge Test

## References

[1] Whiting DR, Guariguata L, Weil C, Shaw J. IDF Diabetes Atlas: Global estimates of the prevalence of diabetes for 2011 and 2030. Diabetes Res Clin Pract [Internet]. 2011;94(3):311–21. Available from: http://www.sciencedirect.com/science/article/pii/S0168822711005912. 10.1016/j.diabres.2011.10.029 22079683

[2] Williams R at all. IDF Diabetes Atlas Ninth Edition. International Diabetes Federation. 2019.

[3] Toobert DJ, Hampson SE, Glasgow RE. The Summary of Diabetes Self-Care. Diabetes Care J. 2000;23(7):943–50. 10.2337/diacare.23.7.943 10895844

[4] Anderson RM, et al. Patient empowerment: results of a randomized controlled trial. Diabetes Care. 1995;18(7):943–9. 10.2337/diacare.18.7.943 7555554

[5] Shrivastava SR, Shrivastava PS, Ramasamy J. Role of self-care in management of diabetes mellitus. J Diabetes Metab Disord. 2013;12 (1):14. 10.1186/2251-6581-12-14 Available from: https://pubmed.ncbi.nlm.nih.gov/23497559. 23497559 PMC3599009

[6] Nolan CJ, Damm P, Prentki M. Type 2 diabetes across generations: from pathophysiology to prevention and management. Lancet (London, England). 2011 7;378(9786):169–81. 10.1016/S0140-6736(11)60614-4 21705072

[7] Ballico E. On the X-rank of a points of the tangent developable of a curve in a projective space. Int J Pure Appl Math [Internet]. 2014;97(2):253–62. Available from: http://www.who.int/about/licensing/copyright_form/index.html%0Ahttp://www.who.int/about/licensing/

[8] Bukhsh A, Khan TM, Nawaz MS, Ahmed HS, Chan KG, Lee LH, et al. Association of diabetes-related self-care activities with glycemic control of patients with type 2 diabetes in Pakistan. Patient Prefer Adherence. 2018;12:2377–85. 10.2147/PPA.S177314 30519003 PMC6235006

[9] Dedefo MG, Ejeta BM, Wakjira GB, Mekonen GF, Labata BG. Self-care practices regarding diabetes among diabetic patients in West Ethiopia. BMC Res Notes [Internet]. 2019;12(1):1–7. Available from: 10.1186/s13104-018-4038-6 30961663 PMC6454742

[10] Abate TW, Tareke M, Tirfie M. Self-care practices and associated factors among diabetes patients attending the outpatient department in Bahir Dar, Northwest Ethiopia 11 Medical and Health Sciences 1103 Clinical Sciences. BMC Res Notes [Internet]. 2018;11(1):4–8. Available from: 10.1186/s13104-017-3111-x 30409148 PMC6225698

[11] Stephani V, Opoku D, Beran D. Self-management of diabetes in Sub-Saharan Africa: a systematic review. BMC Public Health. 2018;18(1):1–11. 10.1186/s12889-018-6050-0 30268115 PMC6162903

[12] Mariye T, Tasew H, Teklay G, Gerensea H, Daba W. Magnitude of diabetes self-care practice and associated factors among type two adult diabetic patients following at public Hospitals in central zone, Tigray Region, Ethiopia, 2017. BMC Res Notes [Internet]. 2018;11(1):1–6. Available from: 10.1186/s13104-017-3088-5 29895315 PMC5998566

[13] Kassahun T, Gesesew H, Mwanri L, Eshetie T. Diabetes related knowledge, self-care behaviours and adherence to medications among diabetic patients in Southwest Ethiopia: a cross-sectional survey. BMC Endocr Disord [Internet]. 2016;16(1):28. Available from: 10.1186/s12902-016-0114-x 27381349 PMC4933997

[14] West JD GK. Diabetes self -care knowledge among outpatients at a veterans affairs medical center. Am J Heal Syst Pharm. 2002;59(9):849–52. 10.1093/ajhp/59.9.849 12004464

[15] Fitzgerald JT, Funnell MM, Hess GE, Barr PA, Anderson RM, Hiss RG D, WK. The reliability and validity of a brief diabetes knowledge test. Diabetes Care. 1998;21(5):706–10. 10.2337/diacare.21.5.706 9589228

[16] World Health Organization. Physical status: The use and interpretation of anthropometry: report of a WHO Expert committee. Technical report series 854. Geneva: World Health Organization; 1995. 8594834

[17] Adem AM, Gebremariam ET, Gelaw BK, Ahmed M, Fromsaseifu M, Thirumurugan DG. Assessment of Knowledge, Attitude and Practices Regarding Life Style Modification among Type 2diabetic Mellitus Patients Attending Adama Hospital Medical College, Oromia Region, Ethiopia. Glob J Med Res. 2014;14(7):37–48.

[18] Ayele BH, Mengesha MM, Tesfa T. Predictors of self-care activities of outpatient diabetic residents in Harar and Dire Dawa: A hospital-based cross-sectional study. SAGE Open Med. 2019;7:205031211986564. 10.1177/2050312119865646 31384462 PMC6647214

[19] Goyal N, Gupta SK. Self-care practices among known type 2 diabetic patients in Haldwani, India: a community based cross-sectional study. Int J Community Med Public Heal. 2019;6(4):1740.

[20] Journal E, Vol MS. KNOWLEDGE, ATTITUDES, PRACTICE AND COMPLIANCE OF DIABETIC PATIENTS IN DAKAHLIA, EGYPT. Eur J Res Med Sci. 2015;3(1):40–53.

[21] Aschalew AY, Yitayal M, Minyihun A, Bisetegn TA. Self-care practice and associated factors among patients with diabetes mellitus on follow up at University of Gondar Referral Hospital, Gondar, Northwest Ethiopia. BMC Res Notes [Internet]. 2019;12(1):1–6. Available from: 10.1186/s13104-018-4038-6 31533833 PMC6751591

[22] Ishak NH, Mohd Yusoff SS, Rahman RA, Kadir AA. Diabetes self-care and its associated factors among elderly diabetes in primary care. J Taibah Univ Med Sci [Internet]. 2017;12(6):504–11. Available from: 10.1016/j.jtumed.2017.03.008 31435286 PMC6694907

[23] Wichit N, Mnatzaganian G, Courtney M, Schulz P, Johnson M. Randomized controlled trial of a family-oriented self-management program to improve self-efficacy, glycemic control and quality of life among Thai individuals with Type 2 diabetes. Diabetes Res Clin Pract [Internet]. 2017;123:37–48. Available from: 10.1016/j.diabres.2016.11.013 27918976

[24] Niguse H, Belay G, Fisseha G, Desale T, Gebremedhn G. Self-care related knowledge, attitude, practice and associated factors among patients with diabetes in Ayder Comprehensive Specialized Hospital, North Ethiopia. BMC Res Notes [Internet]. 2019;12(1):1–7. Available from: 10.1186/s13104-018-4038-6 30658687 PMC6339268

[25] Dinesh P, Kulkarni A, Gangadhar N. Knowledge and self-care practices regarding diabetes among patients with Type 2 diabetes in Rural Sullia, Karnataka: A community-based, cross-sectional study. J Fam Med Prim Care [Internet]. 2016 10 1;5(4):847–52. Available from: http://www.jfmpc.com/article.asp?issn=2249-4863. 10.4103/2249-4863.201176 28349003 PMC5353826

[26] Deepa M, Bhansali A, Anjana RM, Pradeepa R, Joshi SR, Joshi PP, et al. Knowledge and awareness of diabetes in urban and rural India: The Indian Council of Medical Research India Diabetes Study (Phase I): Indian Council of Medical Research India Diabetes 4. Indian J Endocrinol Metab [Internet]. 2014 5;18(3):379–85. Available from: https://pubmed.ncbi.nlm.nih.gov/24944935. 10.4103/2230-8210.131191 24944935 PMC4056139

[27] Mukherjee PS, Ghosh S, Mukhopadhyay P, Das K, Das DK, Sarkar P, et al. A diabetes perception study among rural and urban individuals of West Bengal, India: are we ready for the pandemic? Int J Diabetes Dev Ctries [Internet]. 2020; Available from: 10.1007/s13410-020-00821-8.

